# Coaching in self-efficacy improves care responses, health and well-being in dementia carers: a pre/post-test/follow-up study

**DOI:** 10.1186/s12913-016-1410-x

**Published:** 2016-05-04

**Authors:** Lynn Chenoweth, Jane Stein-Parbury, Danielle White, Georgene McNeill, Yun-Hee Jeon, Beverley Zaratan

**Affiliations:** Centre for Healthy Brain Ageing, Faculty of Medicine, University of New South Wales, Cnr. Botany/High Streets, Randwick, NSW 2031 Australia; Faculty of Health, University of Technology Sydney, PO Box 123, Ultimo, NSW 2007 Australia; Alzheimer’s Australia New South Wales, 120 Coxs Rd (Cnr Norton Road), North Ryde, NSW 2113 Australia; Anglican Retirement Villages, Level 2 Century Corporate Centre, 62 Norwest Boulevarde, Baulkham Hills, NSW 2153 Australia; Sydney Nursing School, The University of Sydney, Mallett Street, Sydney, NSW 2050 Australia; Westmead Hospital, 166-174 Darcy Road, Westmead, NSW 2145 Australia

**Keywords:** Dementia, Carers, Coaching, Self-efficacy, Caregiving hassles, Health, Well-being, Goals

## Abstract

**Background:**

Maintaining the health and well-being of family carers of people with dementia is vital, given their potential for experiencing burden associated with the role. The study aimed to help dementia carers develop self-efficacy, be less hassled by the caring role and improve their health and well-being with goal-directed behaviour, by participating in an eight module carer coaching program.

**Methods:**

The study used mixed methods in a pre/post-test/follow-up design over 24 months, with assignment of consented dementia carers to either individualised (*n* = 16) or group coaching (*n* = 32), or usual carer support services (*n* = 43), depending on preference. Care-giving self-efficacy and hassles, carer health, well-being and goal-directed behaviours were assessed over time. Analysis of Variance (ANOVA) was used to compare changes over time and the effects of coaching on carer self-efficacy, hassles and health, using the Univariate General Linear Model (GLM).

**Results:**

All carers were hassled by many aspects of caring at baseline. Participants receiving coaching reported non-significant improvements in most areas of self-efficacy for caring, hassles associated with caring and self-reported health at post-test and follow-up, than did carers receiving usual carer support. Group coaching had greater success in helping carers to achieve their goals and to seek help from informal and formal support networks and services.

**Conclusion:**

The study outcomes were generally positive, but need to be interpreted cautiously, given some methodological limitations. It has been shown, however, that health staff can assist dementia carers to develop self-efficacy in better managing their family member’s limitations and behaviour, seek help from others and attend to their health. Teaching carers to use goal-directed behaviour may help them achieve these outcomes.

## Background

With one in 20 people over 65 and one in five people over 80 having dementia, the challenges and implications for dementia caregiving are significant [1–3]. As the level of community services available to help families is often inadequate to requirements, these informal carers will often need to regretfully seek long-term care for the person with dementia [3–6]. Family carers play an invaluable social role in dementia care and significantly reduce the national expenditure on long-term care internationally [7–9]. In appreciation of the major contribution that family carers make to dementia care, the Australian Government is urgently seeking ways to ensure their ongoing capacity to care [10]. Major national dementia support organisations in member countries of the Convention on the Organisation for Economic Co-operation and Development (OECD), such as the Alzheimer’s Disease Associations, along with these nations’ policies on ageing, stress that family carers’ needs must be identified and addressed in helping them to continue this role [8–13]. Education and support in targeted areas of need can improve the family carer’s capacity to continue caring [14, 15]. Nevertheless, there are health and economic costs for carers associated with the physical and psychological strains of caring for a person with dementia, as well as loss of social networks, social engagement and employment opportunities [16, 17].

### Carer issues

The most frequently cited causes of carer burden are the presence of impaired cognition and troublesome behaviour in the person with dementia, particularly behaviours that make the carer feel frustrated and/or fearful of their own safety [18] and which also threaten the safety of the person with dementia [19]). An issue that can complicate the caregiving relationship is the care recipient’s resistance to assistance with bladder and bowel control, or continence care [20], situations in which both the carer and the person with dementia can feel powerless [21]. Numerous studies have reported links between functional impairment and disturbed behaviour in people with dementia and stress-related health issues in carers [18–20, 22], with stress being higher in co-resident carers [23].

Carers can find their relationship with their care partner becoming unpredictable and strained [5, 24, 25], particularly when they are unable to adapt to the person’s changing cognition, personality and behaviour [26, 27]. Changes to this relationship can be compounded by tensions in relationships with other family members who have less understanding of the illness and its consequences for the carer [28]. Such tensions cause carers to be reluctant to express their own needs even to close family [15]. The consequence of having to deal with actual or perceived loss of family support can have long-term effects on the carer's psychological adaptation [19, 21] and can cause a breakdown in the care situation [22, 29].

While individual carer characteristics can moderate the impact induced by caring for a person with dementia [19, 30], two major influences on carer burden and adjustment include the level and type of care demands and the resources available in the role [5, 31]. Performing a greater numbers of personal care tasks and spending more caring hours over time correlate with higher carer burden [18, 29], and education and support from a range of carer services reduce burden [25]. The particular skills that carers need to learn are how to obtain help in the caring role [25], knowing how to care for the person with dementia, especially in how to prevent and manage disturbed behaviour [4, 22] and how to pay attention to their own needs [31]) including their own health [3, 32]. A consequence of not seeking help before a breakdown in health occurs can be the emergence of depression [20, 33, 34]. The carer’s feelings of control over the situation may function as a mechanism through which perceived physical health influences psychological health [35].

### Self-efficacy for caring

A carer’s physical and psychological health and coping ability can be moderated by the specific domain of their self-efficacy for the caring role [29, 36, 37]. Understanding the underlying processes that influence a person’s ability to adapt positively to the caring role is an important prerequisite to the development of carer support systems [16]. One influential factor is the carer’s belief that they have the capacity to undertake complex tasks in caring for a family member with dementia [19, 38]. Within social cognitive theory, this belief is labelled as self-efficacy, in reference to the development and maintenance of behaviours over time in response to situational demands [38]. Self-efficacy often changes within the individual over time and in response to specific life experiences, such as taking on the carer role in adult life and having to deal with the changing behaviour and abilities in the care partner [39]. In the context of this demanding role, the carer’s personal resources can mitigate the deleterious effects of the stress and burden they feel [19, 40].

In Bandura’s Self-Efficacy for caring model [38], the caring responsibilities are best achieved when they are broken down into manageable goals. By setting manageable goals for each caring responsibility in turn, carers can be inspired to make additional efforts and persist in achieving further goals once they achieve initial success. Success is more likely when they use strategic steps to plan and execute one goal at a time. Using this process the carer gains more confidence to focus on achieving similar, or associated, goals in different situations [40]. Holding the belief that personal behaviours will influence the achievement of goals in the caring role, is one of the key motivators for doing so [41].

### Purpose of the study

The study’s purpose was to evaluate the effectiveness of a community-based coaching program for carers of people with dementia which aimed to help carers:

1. develop self-efficacy for the caring role;
2. be less hassled by the caring role;
3. pay attention to their health needs;
4. improve their health and well-being; and
5. use goal-directed behaviour to identify and meet their needs.

## Methods

### Design

The study used a pre/post-test/follow-up design using mixed methods over 24 months.

### Setting

The study took place in six Australian community-based dementia carer support services associated with Prince of Wales Hospital and Calvary Health Services in south east Sydney, Alzheimer’s Australia New South Wales (AANSW), the Dementia Behaviour Management Service (DBMAS), the Australian Chinese Community Association (ACCA) and the Australian Nursing Home Foundation in northern, western and south-western Sydney.

### Participants

The convenience sample comprised 91 consenting family carers of people with a moderate to severe level of dementia who were new clients of the six recruited carer support services. Carers were required to be adult family members who provided daily care to a person with dementia who was assessed as having a score of 10–23 on the Mini-Mental State Examination (MMSE) [42] (fluent English speakers), or 16–22 on the Rowland Universal Dementia Assessment Scale (RUDAS) [43] (non-fluent English speakers). Carers also needed a nil/low score (0–1 out of 5) on the five item Geriatric Depression Scale [44], to be able to understand spoken English and to give informed consent.

### Participant recruitment

**Fig. 1:**
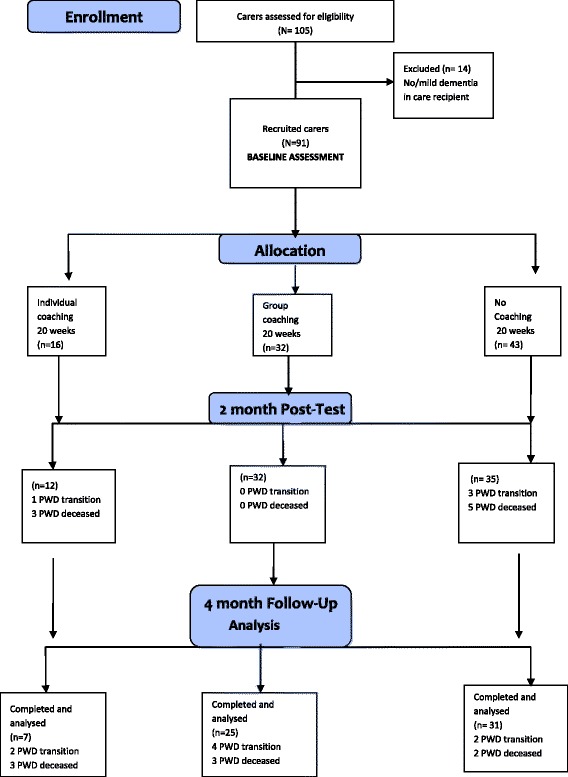
Study protocol

Carer recruitment and participation in the study are shown in Fig. 1, Study Protocol.

Dementia carer support nurses and social workers who were employed by the participating carer support services, but not associated with the study, provided carers with the study information (verbally and in writing) on how to make contact with the lead researcher if they wished to know more about the study. The lead researcher provided the Research Assistants (RA) with details of carers who made contact and expressed an interest to join. While all potential participants met the inclusion criteria of being able to understand English, Chinese carers with limited English skills were provided with translated participant information and consent forms as requested by potential participants. Translation of these forms was conducted by official health translation services, and checked for accuracy by bi-lingual Chinese health workers and consumer group representatives. Three trained health professional research assistants (RA), two of whom were bilingual Cantonese and Mandarin speakers, recruited all participants by accompanying community nurses to carers’ homes during an approved carer support visit and attending carer support groups offered by carer support nurses and social workers. Only 12 out of a possible 105 people with dementia (PWD) were able to give written informed consent to cognitive screening. Written proxy consent was obtained for all PWD, including the 12 people who consented for themselves. Assessment by the RAs of moderate to severe dementia in the PWD signified the carer’s eligibility to join the study; 14 carers were excluded as the care recipient was assessed as having no, or only mild, dementia. The 5-item Geriatric Depression Scale (GDS-5) [44] was administered to eligible carers, since the dementia carer support staff advised that the majority of potential study participants were 65 years or older. Carers were asked to give a ‘yes/no’ response to the GDS-5 which has high sensitivity 0.94 (0.91–0.98), high specificity of 0.81 (0.75–0.87) and a positive predictive value of 0.81 (0.75–0.87) in older adults [44]. Following screening, 91 of 105 potential carer participants were found eligible and invited to join the study by giving written consent.

### Study intervention: Carer Coaching Program

The Carer SE Coaching Program was developed by the lead researcher with assistance of team members, according to the principles advocated by Bandura [45]. The program included eight learning modules focusing on: understanding and developing self-efficacy, or belief and confidence in their capacity to undertake the caring role; developing and practicing self-determined goals; reflecting on achievements and learning to adapt to the caring role; developing self-care and self-help activities; learning person-centred care approaches [45]; reconnecting emotionally with the care recipient; and learning how to obtain assistance with the caring role through informal and formal sources. The three foundations for the coaching program included: training the trainers, tailored learning and mastery and peer support.

#### Train-the trainer approach

Coaching manuals and carer learning manuals and techniques were developed specifically for the study, and were piloted for 12 months prior to the study with five carers of people with Parkinson’s disease having a cognitive impairment and four carers of people with dementia. The pilot materials were revised, reviewed and amended by six dementia carers of different cultural backgrounds, five dementia carer support staff and three team members. When requested, Chinese carers with limited English skills were provided with translated carer manuals that were produced by official health translation services, and approved by Chinese bi-lingual health workers and consumer group representatives. The 14 carer support nurses and four social workers participated in 34 h of group training in carer coaching techniques provided by the Alzheimer’s Australia NSW educator and two expert team members. Once the dementia carer support nurses and social workers were confident to provide carer coaching, they commenced carer coaching under the direct supervision of the educator for the first two coaching sessions. The staff received weekly telephone support from the educator during the first 2 months of the carer coaching program.

#### Individually-tailored learning and mastery

Carer learning activities were tailored to self-identified carer needs and abilities. Semi-structured diaries were used by carers to record and monitor their short and long-term goals and help-seeking behaviour with the assistance of their coach. Carer coaching specifically targeted resource acquisition relevant to the context, skill development and support requirements in achieving goals. During coaching the carers were encouraged to discuss care-related issues, difficulties and successes in caring through experiential and adult learning approaches, helping to develop the foundation for achieving mastery in care techniques. By preference, 16 carers received one-on-one coaching by one of eight community carer support nurses who had received training in the coaching program. Coaching was provided for approximately 1 h each week over 20 weeks in their own homes. Completion of the eight modules ranged between 20 and 30 h, depending on carer learning achievements. Home-based coaching was chosen by these carers, as they were reluctant to access available respite services and/or were unable to attend day-time group coaching sessions because of paid employment. To help these carers participate in one-on-one coaching with minimal disruption, the carer encouraged their care partner to find something interesting to do on their own, such as working/sitting in the garden, watching television, or sorting out their wardrobe. Some carers requested receiving coaching at a time when their care partner normally had a rest in bed, in order to engage in learning uninterrupted. The 32 carers who wished to access available respite services for the PWD received 20 h of group coaching by six different carer support nurses and four social workers working in pairs. Between 6 and 12 carers attended one of six groups for 10 coaching sessions of two hours each, totalling 20 h.

#### Peer support

The coaching staff engaged with each other and the trainers through coaching supervision and telephone support. Carers who participated in group coaching were enabled to connect with other carers for mutual information sharing and support during coaching sessions and through skype/e-mail and telephone. Carers who received coaching in their home were encouraged to attend separate carer support groups offered by the participating community dementia support agencies, and to connect with other carers who were willing to share their skype/e-mail addresses and telephone numbers.

The 43 carers whose expressed need for coaching was less urgent, agreed to receive usual dementia carer support services each week and to remain on a wait list for coaching until follow-up data were collected. These carers were provided with education about dementia, behaviour management, counselling and how to access respite services by carer support service staff not involved in carer coaching; they received carer coaching at the conclusion of the study.

### Study measurement and procedures

Data were obtained by the three RAs who were trained by the lead author in obtaining informed consent and administering study measures to 10 carers who decided not to participate in the study, and their care partners with dementia. The RAs’ inter-rater reliability score calculations reached 0.89 for total item scores on each domain in the Carer Self-Efficacy (SE) Scale [46], the Caregiving Hassles Scale [47] and the Short-Form Heath Survey (SF-12) [48]. Baseline data were obtained prior to carer assignment to individual or group coaching, or to usual dementia support (Fig. 1), with the RAs remaining blind to group allocation. Participant identities were protected by allocating unique code numbers for all recorded data and using confidential codename lists which were retained by RAs in secure file systems. Baseline data included carer demographics- age, gender, English language background, culture, education level, carer status, support from family and friends, total household income (adequate to needs), and daily consumption of alcohol and medicines.

Carer outcome measurement was obtained at baseline (pre-test), 2 months following completion of carer coaching (post-test) and 4 months later (follow-up) and included: Revised Scale for Caregiving Self- Efficacy [46], a modified version of the Caregiving Hassles Scale [47] and the Short-Form Heath Survey (SF-12) [48] (Table 1). Measures were chosen for good validity and reliability with internal consistency using Cronbach alphas of 0.70 and test-retest reliability of 0.50 and above [49, 50].

**Table 1:** Outcome measures

*Outcome measure*	*Domains*	*Scoring and psychometric properties*
Revised Scale for Caregiving Self- Efficacy [46].	Three domains of caregiving self-efficacy: controlling upsetting thoughts associated with caring; responding more effectively to disruptive behaviours; and seeking help in the caring role.	Scoring is on an ordinal scale ranging from 0 (cannot do at all) in increments of 10 through to 100 (certain can do) with higher scores are indicative of greater self-efficacy. Cronbach alphas for all subscales of >0.82–0.85 have been reported.
Caregiving Hassles Scale (modified) [47].	Five subscales: hassles associated with assisting care recipient’s activities of living (ADL) (9 items) and instrumental ADLs (7 items); care recipient’s cognitive status (9 items) and behaviour (12 items); and social network limitations (5 items).	Carers indicate which of the five subscale items occurring during the past week were appraised as a hassle (0 = No, 1 = Yes). Cronbach’s alpha is 0.91 for the full scale and 0.75–0.89 for the subscales, and test-retest reliability is 0.83 for the full scale and 0.66–0.87 for the subscales.
The Short-Form Heath Survey (SF-12) [48, 49].	Eight generic health domains: physical functioning, role general health perceptions, vitality, social functioning, role limitations due to emotional problems and limitations due to physical health problems, bodily pain, mental health, a single-item measure of comparative health.	Scored on Likert scales (1 = Yes, 2 = No), through to 6 response options, depending on the domain. All domain scores are transformed to range from 0–100, with a higher score indicative of a better outcome. Cronbach alpha coefficients above 0.80 are reported, with other psychometric attributes reported as adequate to good.

To assess carer goal-related behaviour at follow-up, one-on-one semi-structured interviews of 30–60 min were conducted with all 91 carers by the RAs in carers’ own homes. Carer verbatim responses were recorded by hand on a paper-based interview booklet. The accuracy of documented carer responses were confirmed by the carer at the conclusion of each interview by reviewing these records. The four carer interview questions included: 1) What were your short and long-term goals? 2) What strategies did you use to achieve your goals? 3) What factors have helped and hindered you in setting and achieving your goals? and 4) What factors helped you with seeking and gaining help in the caring role? The semi-structured diaries used by carers to record their goal activities were reviewed with their permission. Diary entries for the post-test to follow-up period were reviewed to identify any additional recorded information that would help to clarify and confirm the carer’s interview responses.

### Data analysis

Participant demographic data and outcome measurement data were allocated numeric values and entered into SPSS software version 19 [51] by the study RAs. The study’s data manager, blind to study intervention, reversed study measurement scores to fall in the same direction and analysed all data using descriptive statistics. The coaching program’s effectiveness was assessed by a change in scores on the carer’s self-efficacy for caring [46], hassles [47] and health [48]. As carer group sample sizes were small, the Analysis of Variance test (ANOVA) was used to compare changes over time and determine the effects of coaching on carer self-efficacy, hassles and health, using the Univariate General Linear Model (GLM) at 95 % confidence interval [51]. Individual domain and total mean scores were calculated according to the instrument developers’ guidelines [42–47]. Changes in scores from baseline to post-test and follow-up were calculated for all three groups, setting the *P* value at <0.05. The *F* statistic was used to determine if the three study groups’ individual mean scores were significantly different from each other, by comparing the joint effects of carer self-efficacy, hassles and health mean scores. To ensure that these analyses did not produce false positive test results Bonferroni correction was also undertaken, setting the *P* value at 0.025 [51]. Anonymised carer goal data, obtained through interviews and carer goal diary entry reviews were content analysed, guided by Gibb’s framework [52]. Analyses were conducted independently by two of the authors and included: text familiarisation, allocation of text data codes, code building and theme development. These authors then compared data codes to dis/confirm identified themes, and to consolidate and interpret the findings [53]. Any discrepancies were discussed as a team and consensus achieved.

## Results

Of 91 carers enrolled at baseline, 79 remained at post-test and 63 at follow-up. Study drop out occurred by carer choice when the caring role ceased in the home setting because of the death of the PWD (*n* = 12), or the PWD’s transition to an assisted care facility (*n* = 16) (Fig. 1). At baseline participants were caring for a family member with dementia with an average age of 82 years (range 54–93), two thirds of whom were male and with moderately impaired cognition, having an average MMSE score of 19.42 (range 14–28) [42], or a RUDAS [43] score of 15.83 (range 5–28). The characteristics of the 28 exiting carers, including their age and Revised Self-Efficacy Scale [46] and the SF-12 [48] scores, were compared to those who remained. Based on 95 % CIs around these scores, there were no notable differences between those who remained in the study and those who withdrew with the exception of the Vitality domain of the SF-12. Carers who withdrew had a lower mean score of 41.85 (range 40.26–42.21) for Vitality on exiting the study, compared with the mean post-test and follow-up scores respectively for remaining carers: individual coaching (57.3; 62.9); group coaching (68.2; 62.2); and no coaching (54.7; 54.8). As well, the exiting carers’ PWD had reduced cognitive abilities, with an average MMSE score of 17.15, or an average RUDAS score of 14.22.

### Participant characteristics

Carer demographics at baseline are listed in Table 2.

**Table 2:** Carer baseline demographics (*N* = 91)

Age	50–65 (%)	65–74 (%)	75–84 (%)	>85 (%)	
Individual coaching (16)	3 (18.75)	7 (43.75)	6 (37.50)		
Group coaching (32)	11 (34.38)	12 (37.50)	8 (25.00)	1 (3.12)	
No coaching (43)	10 (23.25)	18 (41.86)	10 (23.26)	5 (11.63)	
Gender	M (%)	F (%)			
Individual coaching	4 (25.00)	12 (75.00)			
Group coaching	9 (28.13)	23 (71.88)			
No coaching	8 (18.60)	35 (81.39)			
Cultural background	Australia (%)	Asia (%)	W/Europe (%)	E/Europe (%)	S/America (%)
Individual coaching	8 (50.00)	4 (25.00)	2 (12.5)	1 (6.25)	1 (6.25)
Group coaching	16 (50.00)	12 (37.50)	2 (6.25)	2 (6.25)	
No coaching	18 (41.86)	18 (41.86)	6 (13.95)	1 (2.33)	
English first language	Yes (%)	No (%)			
Individual coaching	11 (68.75)	5 (31.25)			
Group coaching	17 (53.13)	15 (46.87)			
No coaching	22 (51.16)	21 (48.14)			
Education level	Primary (%)	Secondary (%)	Technical (%)	University (%)	
Individual coaching	1 (6.25)	9 (56.25)	4 (25.00)	2 (12.50)	
Group coaching	2 (6.25)	11 (34.38)	10 (31.25)	9 (28.12)	
No coaching	1 (2.33)	15 (34.88)	21 (48.84)	6 (13.95)	
Current employment	Yes (%)	No (%)			
Individual coaching	2 (12.50)	14 (87.50)			
Group coaching	3 (9.38)	29 (90.62)			
No coaching	17 (39.53)	26 (60.47)			
Sole carer	Yes (%)	No (%)			
Individual coaching	11 (68.75)	5 (31.25)			
Group coaching	22 (68.75)	10 (31.25)			
No coaching	30 (69.77)	13 (30.23)			
Family/friend support	Yes (%)	No (%)			
Individual coaching	10 (62.50)	6 (37.50)			
Group coaching	21 (65.62)	11 (34.36)			
No coaching	31 (72.09)	12 (27.91)			
Income adequate	Yes (%)	Partially (%)	No (%)		
Individual coaching	7 (43.75)	7 (43.75)	2 (12.50)		
Group coaching	12 (37.50)	16 (50.00)	4 (12.50)		
No coaching	25 (58.14)	14 (32.56)	4 (9.30)		
Glasses of alcohol/day	0	1–2	3 or more		
Individual coaching	9 (56.25)	3 (18.75)	4 (25.00)		
Group coaching	19 (59.38)	10 (31.25)	3 (9.37)		
No coaching	22 (51.16)	18 (41.86)	3 (6.98)		
Number of medicines	0	1–3	4–7	8 or more	
Individual coaching	5 (31.25)	10 (62.50)	1 (6.25)		
Group coaching	11 (34.38)	15 (46.88)	6 (18.75)		
No coaching	21 (48.84)	15 (34.88)	7 (16.28)		

Baseline: Individual coaching (*n* = 16); Group coaching (*n* = 32); No coaching (*n* = 43)

Percentages (%) rounded up to two decimal points

### Carer outcomes

Despite the small sample sizes in each group, the data were predominantly normally distributed with very few departures from normality.

#### Caregiving Self-Efficacy (SE)

Prior to coaching, across three domains of SE [46] the non-coaching group reported higher (better) mean scores than carers receiving individual coaching and carers involved in group coaching in Seeking Help from Others: 290.0 compared with 191.1 (individual coaching), and 234.2 (group coaching) and Managing Behaviours 354.0 compared with 311.6 (individual coaching) and 343.7 (group coaching). All three groups had similar mean scores for Controlling Negative Thoughts Associated with Caring (360.2, 354.2 and 355.3 respectively). Comparative data of a sample of 145 North American men and women from one major city who were caring for a family member or close friend with dementia [46] indicated similar levels of self-efficacy for caring. While the total mean score for Caregiving SE was not significantly improved with coaching over time (Table 3), the individually coached carers were more confident to seek help and respite for their care partner than the group-coached carers (Table 4). Non-significant improvements occurred for carers involved in both individual and group coaching in SE for Managing Behaviours and Controlling Negative Thoughts Associated with Caring over time. These changes compare favourably with the non-coached group, whose mean Caregiving SE scores reduced (Tables 3 and 4).

**Table 3:** Mean Scores: Group x Time - Caregiving Self-Efficacy, Caring Hassles and Carer Health

	Pre coaching (*n* = 91)	Post coaching (*n* = 79)	Follow-up (*n* = 63)	*p*	F-
Caregiving Self-Efficacy					
Possible total score range 0–500, higher score is better				Group x Time	
Non coaching	335	285	291	1.20	0.00
Individual coaching	286	343	371	0.37	0.98
Group coaching	311	352	369	0.52	0.47
Caring Hassles					
Possible total score range 0–30, higher score is worse				Group x Time	
Non coaching	10.1	10.6	9.0	0.95	0.01
Individual coaching	14.2	13.2	8.5	0.58	0.38
Group coaching	9.2	7.2	6.2	0.73	0.15
Carer Health					
Possible score range 0–100, higher score is better				Group x Time	
Non coaching	63.70	63.16	61.35	0.99	0.00
Individual coaching	62.24	71.81	77.78	0.45	0.52
Group coaching	66.88	72.85	74.43	0.65	0.23

**Table 4:** Changes in Caregiving Self-Efficacy: Non, Individual, Group Coaching

Caregiving self-efficacy							
Domain	Stage	Non coaching		Individual coaching		Group coaching	
		Mean	*p*	Mean	*p*	Mean	*p*
Seeking help/respite	Pre-test	290.0		191.1		234.2	
	Post-test	205.3	0.28	321.7	0.12	266.6	0.82
	Follow-up	203.8	0.33	343.3	0.63	299.8	0.23
Managing behaviours	Pre-test	354.0		311.6		343.7	
	Post-test	323.5	0.79	333.3	0.46	387.7	0.21
	Follow-up	330.3	0.91	383.3	0.15	393.8	0.13
Controlling negative thoughts	Pre-test	360.2		354.2		355.3	
	Post-test	325.3	0.74	372.5	0.79	400.3	0.78
	Follow-up	338.5	0.94	385.0	0.32	415.2	0.25

Pre-Test (*N* = 91), Post-Test (*N* = 79), Follow-Up (*N* = 63)

Three domains contain five items each. Score: 0 (cannot do at all) in increments of 10 through to 100 (certain can do). Range: 0 to 500

#### Caring Hassles

At baseline most of the carers reported being hassled by many caring-related issues, including their care partner’s Behaviour (mean 4.23) and Cognitive Status (mean 3.9), and the need to assist the PWD with Activities of Living (mean 1.13) and Instrumental Activities of Living (mean 1.33). Non-coaching group carers continued to be hassled mainly by the PWD’s Cognitive Status at post-test and follow-up (mean 3.4, 3.1 respectively) and Behaviour (mean 3.5, 3.4 respectively). Table 3 shows a reduction in the mean scores for caregiving hassles of all groups over time, with the greatest reduction occurring in carers receiving individual coaching. While most of the carers who participated in individual coaching remained hassled by the PWD’s Cognitive Status at post-test (mean 4.7), this declined significantly by follow-up (mean 2.3). To a lesser extent, hassles associated with Cognitive Status reduced for group-coached carers at follow-up. The other notable reduction in hassles for individually-coached carers was for Behaviour (mean 2.5) (Table 5).

**Table 5:** Changes in Caring Hassles: Non, Individual, Group Coaching

Caregiving Hassles							
Hassles subscales	Stage	Non coaching		Individual coaching		Group coaching	
		Mean	*p*	Mean	*p*	Mean	*p*
Behaviour	Pre-test	3.7		5.1		3.9	
	Post-test	3.5	0.98	4.2	0.85	2.7	0.69
	Follow-up	3.4	0.83	2.5	0.08	2.6	0.62
Activities of Daily Living (ADL)	Pre-test	1.0		1.6		0.8	
	Post-test	1.7	1.20	2.1	1.89	0.9	1.10
	Follow-up	1.3	1.10	1.7	1.00	0.7	0.96
Social network	Pre-test	0.5		0.6		0.6	
	Post-test	0.5	1.00	0.9	1.10	0.6	1.00
	Follow-up	0.1	0.24	0.6	1.00	0.6	1.00
Instrumental ADL (IADL)	Pre-test	1.3		1.9		0.8	
	Post-test	1.5	1.10	1.3	0.71	0.9	1.10
	Follow-up	1.1	0.89	1.4	0.79	0.7	0.96
Cognitive status	Pre-test	3.6		5.0		3.1	
	Post-test	3.4	0.89	4.7	0.90	2.3	0.69
	Follow-up	3.1	0.80	2.3	0.04	1.6	0.50

Pre-Test (*N* = 91), Post-Test (*N* = 79), Follow-Up (*N* = 63)

Subscales: Hassles associated with the care-partner’s behaviour (12 items), Assistance in basic ADL (9 items), Caregiver’s social network (5 items), Assistance in IADL (7 items), and Care-partner’s cognitive status (9 items). Score: N0 = 0, YES = 1

#### Carer health

At baseline all carers’ SF-12 [48] mean scores for every area of self-rated health were lower (75.2) than mean scores of Australian adult carer SF-36 normative data (84.2) [54]. The study carers’ health was more impacted by the caring role than the health of carers of children with physical and/or intellectual disabilities and carers of adults with physical disabilities [54]. As shown in Table 3, all three carer groups had similar mean baseline health scores, which improved over time for both of the coached carer groups. By follow-up carers who participated in individual coaching consistently reported higher, non-significant mean scores for: Physical Functioning (mean 84.3), Role Physical (mean 78.6), Bodily Pain (mean 86.1), General Health (mean 74.9), Vitality (mean 62.9), Social Functioning (mean 89.3), Role Emotional (mean 72.4) and Mental Health (mean 73.7) (Table 6). Carers participating in group coaching reported non-significant increases in health scores from baseline, particularly for Role Physical (mean 72.0), and also for Bodily Pain (mean 75.8), General Health (mean 64.8), Vitality (mean 62.2), Social Functioning (mean 80.7), Role Emotional (mean 77.8) and Mental Health (mean 75.4). Nevertheless, the non-coached group slightly improved their follow-up scores for Bodily Pain (from 62.8 to 64.8) and Social Functioning (from 63.6 to 68.4) (Table 6).

**Table 6:** Changes in carer-rated health: Non, Individual, Group Coaching

SF-36 Health Survey							
Sub-scales	Stage	Non coaching		Individual coaching		Group coaching	
		Mean	*p*	Mean	*p*	Mean	*p*
Physical functioning	Pre-test	72.6		73.2		76.0	
	Post-test	72.9	1.00	82.7	0.64	77.8	0.80
	Follow-up	70.9	1.20	84.3	0.74	79.5	0.78
Role limitations Physical health	Pre-test	58.7		55.0		60.7	
	Post-test	54.4	1.23	63.6	0.23	72.1	0.06
	Follow-up	50.0	1.30	78.6	0.01	72.0	1.00
Bodily pain	Pre-test	62.8		72.7		68.9	
	Post-test	64.1	1.00	73.9	0.89	70.8	0.82
	Follow-up	64.8	0.99	86.1	0.69	75.8	0.53
General Health	Pre-test	66.5		65.3		63.9	
	Post-test	58.2	1.42	74.5	0.67	63.8	0.99
	Follow-up	60.6	1.26	74.9	0.59	64.8	0.88
Vitality	Pre-test	54.8		48.3		50.6	
	Post-test	54.7	1.00	57.3	0.57	68.2	0.22
	Follow-up	54.8	1.00	62.9	0.28	62.2	0.69
Social functioning	Pre-test	63.6		67.5		71.1	
	Post-test	68.4	0.90	84.1	0.27	84.9	0.45
	Follow-up	68.8	0.88	89.3	0.18	80.7	0.58
Role limitations Emotional health	Pre-test	61.6		57.8		72.4	
	Post-test	62.7	1.00	69.7	0.87	73.4	0.66
	Follow-up	52.1	1.22	72.4	0.69	77.8	0.59
Mental Health	Pre-test	69.2		58.1		71.4	
	Post-test	69.9	1.11	68.7	0.44	75.1	0.77
	Follow-up	68.8	1.14	73.7	0.32	75.4	0.76

Pre-Test (*N* = 91), Post-Test (*N* = 79), Follow-Up (*N* = 63)

Range: 0 (lowest level of functioning) to 100 (highest level of functioning)

### Carer goal achievement

Reported carer goal achievements varied in scope across all three groups, but were more positive for the coaching group carers, with many long and short-term goals being achieved using strategic approaches. At post-test five of the 12 carers receiving individual coaching identified some difficulties in working with their community nurse coaches to decide on short-term goals and how these could be achieved, mainly because they did not discuss desirable goals with family and friends, or other carers. By contrast, 28 of the 32 carers engaged in group coaching reported success and satisfaction in goal achievement for themselves and their care-partner, as a result of sharing ideas with other carers and because of the goal development activities conducted during group learning. In contrast, at post-test only six out of 35 carers who did not participate in coaching attempted to use their goal diary to identify and achieve self-determined goals. The following themes were common for all carers receiving coaching, whose confidentiality was maintained by allocating unique de-identified codes to their verbatim interview responses.

#### Understanding the reasons and better managing behaviour

A key carer goal in supporting their own health and well-being was to understand the reasons for their family member’s behaviour as the first step to better managing the behaviour. This was achieved by learning to appreciate the person more and understanding how the dementia disease process was affecting their abilities and behaviours.

*“I would like to better manage my husband’s forgetfulness by asking where he had placed it last, help him find it and not get angry about the situation”*(CI10)

Since gaining knowledge of how to reduce the stimuli that tended to trigger some behaviours was common for all carers, achieving this goal gave carers a great deal of satisfaction and feelings of well-being.

*“I am able to create strategies to prevent him from becoming agitated when shopping”* (PWC07)

#### Seeking support from others

While all carers were offered advice and assistance to access respite care so that they could attend carer support groups, engage with the coaching program and take time out for social activities, at least half of all carers elected not to take this opportunity initially, citing an unwillingness to leave their care partner with *‘strangers’.* As well many carers believed that their care partner ‘*would be upset if they were left in the care of others’*. Carers whose goal was to seek support from others, including extended family, were happy to find that this support was accepted by their family member and it helped them to have a more objective approach to their role.

*“It has helped me to speak out about my issues to people who understood me.*

*The fact that I had someone to talk to when I felt like I didn’t know what was going on made it a lot easier for me”* (CACA11)

*“I now go out with grandchildren for a change of environment”*(CACA23)

Over time, the majority of these carers reported positive effects from setting out strategies to seek support in the caring role. Help-seeking included “*seeking emotional/social support”*(CG25), “*maintaining links with a larger social network”* (CG30) and “*seeking respite care*” (C102), particularly for carers attending group coaching. Control group carers, on the other hand, continued to express feelings of stress and despondency throughout the study in regard to gaining support from others.

#### Feeling more confident in the caring role

Carers reported that focusing on self-determined goals helped them to feel more confident in the caring role. The responses included being able to cope more effectively and to accept guidance in caring.

*“By creating goals it has made me feel strong and confident as I am able to create ways of convincing my care partner to do what I want him to do. By allowing health services…to assist with the way I care for my partner, has helped me tremendously”*(CG22)

#### Engaging in health maintenance

Carers reported that one of the positive effects of goal–focused behaviour and learning to develop SE for caring was in focusing on their own and their care partners’ health and wellbeing. Engaging in healthy activities included attending to normal life activities uninterrupted by their family member.

*“It definitely has improved my health and well- being, being on my own for just a couple of hours while my husband goes to respite care make me feel free. It makes me feel I can do what I can’t do such as cleaning, ironing and shopping when he is around. Even if it’s just going out to buy vegetables and having coffee makes me feel that I had a wonderful day”* (CI06)

*“One of my goals was for my husband to attend to his appointments in which I had to constantly convince him to go. Due to the fact that he had listened to me he has now lost one kilo and he has his diabetes in control”* (CG02)

### Enablers to achieving goals

Two themes emerged in relation to enablers of goal achievement: commitment and acceptance. A strongly-held sense of commitment enabled carers to achieve the goal of maintaining close connection with their relative, despite continuing deterioration and the difficulties faced each day, which was an important well-being factor.

*“I want to keep the promise I made fifty eight years ago to love my partner through sickness and in health, richer and poor”* (CI14)

*“Making him happy and appreciate where he is coming from” (*PWCA02)

Accepting the negative effects caused by dementia, especially in the capabilities of their family member, made it less difficult for carers to achieve their goals. These included feeling contented with the difficult caring role, the situation they found themselves in and learning how to remain calm.

*“I redirect my anger towards the sickness not towards my Care Partner. I try to understand that the sickness is making him act wrongly”* (CG11)

*“Just trying to get along on a day to day basis, be a happy person and stay well”* (CACA11)

### Barriers to achieving goals

Feelings of guilt and their family member’s limited abilities were identified as key themes which created barriers for carers in achieving their goals. Carers generally expressed feelings of guilt when they decided to prioritise their own needs, rather than their care partner’s needs, mainly because of the weight of responsibility as a sole carer, and if things went wrong for their partner when accepting help from others.

*“My husband’s needs, when I go on a holiday, I feel worried and I feel that I am responsible for anything wrong that would happen to him. I can’t enjoy my holidays because I feel guilty and I feel worried”* (CI07)

Care partner memory loss and failing concentration were limitations that created barriers to the carers’ goal of helping their partner to be more independent. Coming to terms with their family member’s limitations in being able to independently attend to activities of daily living, such as bathing and toileting, was one of the most difficult aspects of the caring role.

*“…the sickness itself and their behaviour of constantly forgetting, this means I can’t achieve my goal of getting him to do more for himself”* (CG11)

## Discussion

Carer coaching achieved the aims of helping most of the family carers to develop self-efficacy in the caring role, by responding less negatively and better managing disruptive behaviours in their family member with dementia (PWD) and seeking formal and informal help with care. As well, carers receiving coaching more readily adopted goal-directed behaviour to identify and meet their own needs, including their health and well-being, as well as the health of their family member with dementia. These achievements also related to feelings of being supported, enabled and empowered in the caring role. From poor baseline scores the carers’ confidence with managing the behaviour of their care partners surpassed international comparative data [46] at post-test and follow-up. While improvements over time in the carers’ self-efficacy, hassles and health scores did not improve significantly, such improvements in responses to the caring role may, nevertheless, be clinically significant.

Similar to international study findings, at interview study carers reported having high levels of stress, despondency and exhaustion in the caring role prior to coaching [3–5]. They reported having low self-efficacy for caring responsibilities [22, 27–30] and that their health and well-being were being negatively affected by caring [3, 34–37]. Carers had lower levels of self-reported health at baseline than Australian carers of adults with intellectual and/or physical disabilities and carers of adults in poor health [54]; they reported being unable to think about their own needs while concentrating on their immediate and many caring responsibilities. Their focus was on how to cope with new responsibilities that arose for them owing to the PWD’s failing abilities and behaviour. Some carers indicated that they did not have the support of family and friends in this role, while others reported their income was insufficient to their needs and to pay for community support services, such as respite care. At least half of all carers reported consuming one or more glasses of alcohol each day and taking regular medicines for stress related conditions, which may indicate non-effective coping.

A carer issue reported in the literature is the safety, health and well-being of a family member with dementia [4, 15, 19, 28]. This was also a significant concern for all carers prior to coaching and one which impacted on the carer’s own health. Some of the first goals established by carers were associated with paying attention to these health concerns. The carers participating in group coaching were more willing to seek help outside of the immediate family for both themselves and their care partner, particularly for help with maintaining the health and safety of their care partner. Similar to other carers in receipt of targeted support [16, 25, 31, 32], these carers were willing to investigate and make use of a wider range of community based services, were more likely to accept the negative effects associated with dementia and more readily worked on strategies that would assist with adapting to the care partner’s cognitive decline. While initially reluctant to participate in group learning activities, the carers who chose to receive individualised coaching in their own homes were more open to seeking advice and support from other carers as coaching progressed, and were more willing to attend dementia support group offered by dementia health services and Alzheimer’s Australia. Compared with the non-coaching group, the majority of carers who received individual coaching were able to frame their caring issues and responsibilities more positively, which suggests that developing self-efficacy for caring can help in taking better control of the complex caring role [21].

Following coaching carers were also more content with their situation, less hassled by their care partner’s behaviour and were more willing to seek help and support in the caring role when required. This was a positive outcome of coaching, since an issue for respite services and respite referral agencies is to convince carers that having time away from the caring role will ultimately help them to sustain their health and caring abilities over the longer term [17, 20, 26]. Coaching was a catalyst for having Chinese carers, in particular, accept and make use of respite services. These carers initially expressed reluctance to access respite services because of the cultural stigma associated with having a family member with dementia, the tradition of forbearance when faced with difficult family issues and strong family obligations to care for older members [55]. In contrast to Chinese carers of people with dementia in the USA [56] and Hong Kong [57], these strongly-held views were relaxed during the coaching program when Chinese carers accepted that their own health was a paramount factor in their ability to continue in the caring role.

Many of the health related goals that carers set for themselves were achieved through stress reduction techniques learnt during the coaching program and by giving themselves permission to let go of insoluble issues. However, more ambitious goals, like taking a holiday and getting their care partner to be more independent in self-care, were sometimes thwarted by the negative reaction of their care partner to these plans. As previously identified in the literature, the lowered cognitive abilities of the care partner to achieve self-care, and/or the guilt experienced by the carer themselves when focusing on their own needs, were factors associated with goal revision [12, 15, 30]. For some exiting carers, however, the decision to seek residential aged care support services for their care partner was made in light of the goals to reduce their stress levels and to regain their health. International research on carer decisions regarding seeking external support suggests that the carer’s own deteriorating health is often the main factor in help-seeking [4, 31]. Consequently, if the carer’s health was continuing to deteriorate, despite the assistance and education received, their participation in coaching may not have been the catalyst for the decision to seek supported long-term care services for the PWD.

Carers involved in group coaching had far greater opportunities to interact with their peers than individually coached carers, which allowed frank sharing of experiences, feelings and effective caring techniques, more so than carers who attended regular support groups. This was a particularly satisfying experience for the Chinese speaking carers who had hitherto found it difficult to accept and adapt to mainstream dementia support service opportunities. As previously reported [23, 26, 27] carers attending group coaching also found it easier to develop goal-focused behaviour and were more successful in achieving short and long-term goals, compared with carers who received individualised coaching. These different outcomes may have related to the quality of carer education and support and/or peer-support. As all carer support personnel were assessed as having the necessary carer coaching knowledge and skills prior to implementing the program, were supervised during the initial coaching sessions and were provided on-going advice by telephone, differences in the quality of carer support were minimised. Group-based learning and peer-support may, therefore, be of greater benefit to carers confronting similar issues.

These study findings highlight the important role that health staff can play in providing dementia carers with tailored education and training for the caring role, in providing advice and training in understanding and responding therapeutically to the care partner’s behaviour, and in developing the confidence and skills in devising and executing clear and achievable goals for self-care and help-seeking. Since group learning was more successful in assisting carers to achieve their goals, and superior to non-coaching support services, group carer support is recommended. Nevertheless, carer support groups are most effective when they are sufficiently flexible in catering to individual carer needs and capabilities, while offering education and support commonly required by all members [16, 32]. Since each carer’s needs and circumstances are unique it is, therefore, important that dementia support services are tailored to individual carer needs at the group and individual level. For dementia carers who are unable, or unwilling, to join targeted group education, coaching and support programs, dementia services need to give carers the opportunity to receive one-on-one coaching until they gain the confidence to access group programs. To offer this level of flexibility, dementia support services must ensure that their staff are suitably educated and supervised in all forms of coaching, especially in facilitating the development of carer self-efficacy in the caring role.

### Strengths and limitations

One successful aspect of the carer coaching program was that at the end of the study the majority of carers who had received coaching decided to meet regularly with other carers for mutual support and were very satisfied with the coaching support they had received. The benefits arising for carers who hitherto had not been willing to accept carer support and respite services through available support agencies, included their realisation that caring responsibilities can be shared with others outside the family without any deleterious effects to the PWD. Carers receiving individualised coaching were, subsequently, far more willing to join formal carer groups convened by the participating carer support agencies by the end of the study and also to accept respite care.

Another important outcome of the study was the ongoing coaching training being passed on to colleagues by the nurses and social workers who mastered these skills during the study. The carer coaching program has been embedded in the carer support offered by these services, enabling further skill training for their staff and opportunities for over 400 carers who have subsequently accessed their services.

While the subsequent outcomes of this process are likely to be favourable for service providers and carers, the study findings are limited to a relatively small convenience sample which may not be representative of all community-based family carers of people with dementia. Carers of people with mild dementia were excluded from the study and only a quarter of the carers’ family members had advanced dementia. Participants also resided in a major Australian city with access to community-based support services and subsidised respite care. The study findings are, therefore, of limited value in making recommendations for educating carers who are facing a new diagnosis of dementia in a family member and for carers who face insurmountable caring challenges occurring in the later stage of the disease, including having access to respite services that are able to accommodate significant cognitive limitations in the PWD.

A major study limitation was the non-random allocation of equal numbers of carers to the three study groups, and no adjustment for carer baseline characteristics prior to group allocation. The study sample size in each group was insufficient to show a statistically significant improvement in self-rated health and self-efficacy for caring. To detect a mean difference of a change in 30/100 points and a standard deviation of 25.0 (i.e., an effect size of 0.64) in the SF-12 baseline to follow-up total scores, a sample size of 50 in each study group at follow-up would be needed to detect a clinically important difference with 80 % power [58]. While there was an improvement in carer reported health, self-efficacy and hassles for some aspects caring, the non-randomly allocated study groups and small sample size limit generalisation of the study findings.

Relying on a single measure of carer health with the self-report SF-12 scale and carer responses during interviews on goal behaviour, also represents a study limitation. Carer health, well-being and coping abilities are broad concepts that require deeper investigation than occurred in this study. This is an area of interest that the authors intend investigating in further studies of carer support. While the study results were mainly positive, in light of the noted study limitations they need to be interpreted cautiously.

## Conclusions

The carer coaching program offered to carers by trained community nurses and social workers, either in their own homes or in group sessions in community centres, moderately improved their self-efficacy for caring, most areas of perceived health, goal-directed behaviour and confidence in seeking help from others in the caring role. In contrast, many of the 31 remaining carers who were supported by usual dementia carer support services and received no coaching, remained hassled by aspects of caring, were reluctant to seek help in the role and most areas of their health either deteriorated or remained unchanged. The findings concur with the positive outcomes arising from other carer programs that have focused on building carer knowledge, confidence and health [36, 41]. Since group coaching was more likely to help carers achieve their goals and to seek help from informal support networks and formal services, health care staff are advised to assist dementia carers to access support groups/services that provide these incentives. They are especially encouraged to identify and address carer’s needs for further education, skill development and assistance in the caring role, since developing self-efficacy for caring will most likely prevent premature and stress-provoking admission of their care partner to institutional care. Carer interviews confirm the relevance of learning techniques for developing self-efficacy for caring in goal achievement and in this respect, it is recommended that the coaching program would be beneficial to investigate with other groups of carers in need of support.

## Declarations

### Ethical approval and consent to participate

Ethical approvals for the study were obtained from: the human research ethics committees of the University of Wollongong, a lead ethics committee for all regions in the state of NSW (HEC/10/WGONG/134); the University of Technology (UTSHREC 2011-258R; 2012-258R1); Prince of Wales Hospital (SSA/11/G/017); Calvary Health Services (SSA/11/STG/44); and Northern Sydney/Central Coast Local Health District (HEC/10/12/134A). All study participants provided written approval to participate and where people with dementia lacked capacity, proxy consent from family carers was obtained.

### Consent to publish

The study participants have given written approval for non-identifiable study data to be published in summary form.

### Availability of data and materials

The study data are archived at the University of Technology Sydney, Australia, and will not be available for sharing owing to sensitive issues raised by study participants which they did not want shared with anyone apart from the data collectors and the chief investigator. The University of Technology Sydney holds copyright of the carer coaching learning resources and training manuals which are available on issue of a license under contract.

## Abbreviations

AANSW: Alzheimer’s Australia New South Wales
ACCA: Australian Chinese Community Association
ANOVA: analysis of variance test
Caregiving SE: caregiving self-efficacy
DBMAS: dementia behaviour management service
GLM: Univariate General Linear Model
PWD: person with dementia
MMSE: mini-mental state examination
RA: research assistant
RUDAS: Rowland Universal Dementia Assessment Scale
SF-12: short-form heath survey
